# Draft Genome Sequence of Enterobacter cloacae 3D9 (Phylum Proteobacteria)

**DOI:** 10.1128/MRA.00902-18

**Published:** 2018-10-25

**Authors:** Christopher R. Dumigan, Gregory E. Perry, K. Peter Pauls, Manish N. Raizada

**Affiliations:** a Department of Plant Agriculture, University of Guelph, Guelph, Ontario, Canada; University of California, Riverside

## Abstract

Presented here is the draft genome sequence of Enterobacter cloacae 3D9. This candidate seed endophyte was isolated from Zea nicaraguensis. The genome contains 4,653,375 bp in 28 contigs.

## Abstract (precis)

Presented here is the draft genome sequence of Enterobacter cloacae 3D9. This candidate seed endophyte was isolated from Zea nicaraguensis.

## ANNOUNCEMENT

Endophytes are microbes that exist inside plants without causing disease (1). Endophytic species, such as Enterobacter spp., likely play important roles in plant microbiomes, as many have been found to act as plant growth-promoting bacteria in diverse crops, such as maize, rice, and tomatoes (2, 3). There are a variety of mechanisms by which Enterobacter species can promote plant growth, such as the solubilization of phosphate (4) or fixation of atmospheric nitrogen (5).

Grasses such as maize, wheat, and rice are the world’s three most important food crops, but these grasses require large amounts of nitrogen fertilizer. There is incentive to find alternative ways to provide usable nitrogen to these crops, and the use of microorganisms is currently an attractive research target. Recently, a screen of 75 candidate endophytes isolated from diverse wild and domesticated maize genotypes was performed to identify strains that could increase the biomass of annual ryegrass, a maize relative, in a nitrogen-free medium (6). The candidate endophyte Enterobacter cloacae 3D9 consistently promoted plant biomass in a nitrogen-free medium. The growth-promoting mechanism of action is unknown, but possibilities include nitrogen fixation, recycling of nitrogen from senescing plant tissue, or scavenging of secreted nitrogen metabolites (6, 7). Here, we present the draft genome sequence of E. cloacae 3D9 in hopes of further understanding its mechanism of action.

E. cloacae 3D9 was isolated from surface-sterilized seeds of Zea nicaraguensis in 2011 (8). For genomic DNA isolation, a single colony from an LB plate was used to inoculate an overnight LB culture at 30°C with shaking at 200 rpm. A bacterial genomic DNA isolation kit (catalog number 17900; Norgen, Thorold, ON, Canada) was used to extract genomic DNA from the overnight culture. The genomic DNA (1 ng) was used to create a paired-end library using a Nextera XT DNA library prep kit (Illumina, San Diego, CA). Sequencing was carried out using an Illumina MiSeq instrument with 50 pg of 3D9 genomic DNA, which yielded 10,039,290 reads with an average length of 251 bp for a 542-fold coverage. In total, 10,032,951 reads remained after trimming low-quality reads. *De novo* assembly was performed using CLC Genomics Workbench (v.10.0.1; Qiagen) and resulted in 23 contigs (minimum length, 1,164 bp; maximum length, 935,739 bp; *N*_50_, 396,804 bp). The assembled genome had 4,653,375 bp and a GC content of 56.2%.

The Rapid Annotations using Subsystems Technology (RAST) server was used to annotate the genome sequence (9). Using KmerFinder 3.0, the species was determined to be E. cloacae (10, 11), with 98.6% nucleotide similarity to Hoffman complex IV (12). E. cloacae IV 3D9 contains 4,262 predicted protein-coding genes and 74 noncoding RNAs. Neither BLAST nor RAST searches identified a nitrogen fixation operon in the genome, suggesting that the genes are located on a plasmid or that there is an alternative mechanism of plant growth promotion under nitrogen-limited conditions.

### Data availability.

The whole-genome shotgun project was deposited at DDBJ/EMBL/GenBank under the accession number QKSC00000000. The version described in this paper is version QKSC01000000. Raw Illumina reads are available in the SRA under accession number SRR7341760.
